# Chikungunya virus TF inhibits SNX27-mediated endocytic recycling of GLUT1

**DOI:** 10.1128/spectrum.04137-25

**Published:** 2026-05-13

**Authors:** Shengnan Wang, Dan Zhang, Leiliang Zhang

**Affiliations:** 1 Department of Clinical Laboratory Medicine, The First Affiliated Hospital of Shandong First Medical University & Shandong Provincial Qianfoshan Hospital 66310 , Jinan, Shandong, China; 2 Department of Pathogen Biology, School of Clinical and Basic Medical Sciences, Shandong First Medical University & Shandong Academy of Medical Sciences 518873, Jinan, Shandong, China; Chinese Academy of Sciences, Wuhan Institute of Virology, Wuhan, China

**Keywords:** CHIKV, TF, GLUT1, glucose, SNX27

## LETTER

**Fig 1:**
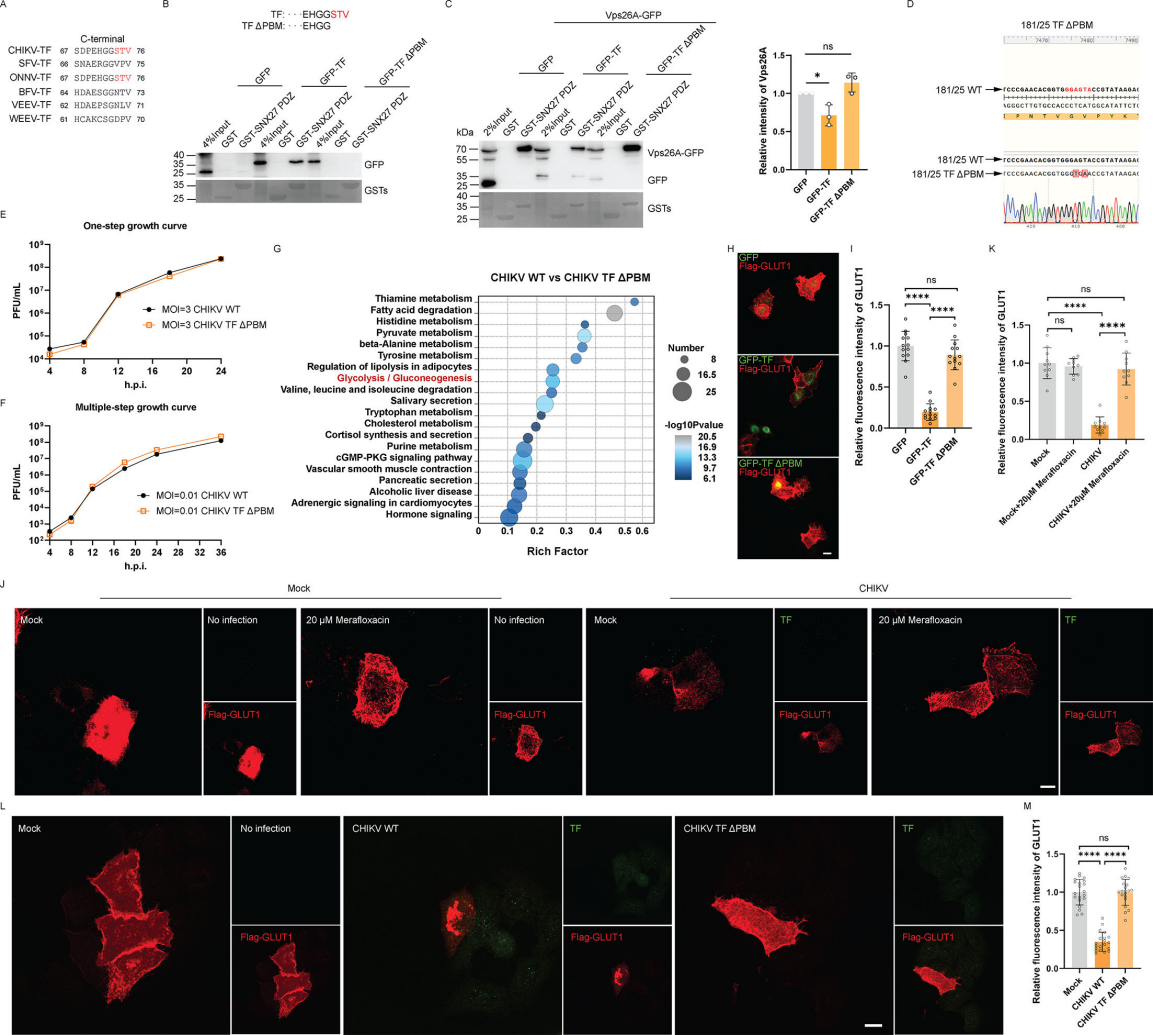
CHIKV TF inhibits SNX27-mediated GLUT1 endocytosis through a PDZ binding motif. (**A**) CHIKV TF contains a PDZ-binding motif. TF proteins from CHIKV, Semliki Forest virus (SFV), O'nyong-nyong virus (ONNV), Barmah Forest virus (BFV), Venezuelan equine encephalitis virus (VEEV), and Western equine encephalitis virus (WEEV) were aligned. The C-terminal STV in TF from both CHIKV and ONNV, highlighted in red, is identified as a PDZ-binding motif. (**B**) TF interacts with SNX27 PDZ. Lysates from HEK293T cells transfected with constructs expressing GFP-tagged TF and TF ΔPBM were subjected to pulldown using GST-SNX27 PDZ or GST. Input represents 4% of the total cell lysates. (**C**) TF inhibits the interaction between Vps26A and SNX27 PDZ. Lysates from HEK293T cells transfected with constructs expressing GFP-tagged TF, TF ΔPBM, and Vps26A-GFP were pulled down with GST-SNX27 PDZ or GST. Input represents 4% of the total cell lysates. The relative intensity of Vps26A and SNX27 PDZ was normalized by quantifying three repeated experiments using ImageJ. Statistical significance was determined using unpaired two-tailed multiple *t*-tests. ns, *P* > 0.05; *, *P* < 0.05. (**D**) Validation of CHIKV 181/25 TF ΔPBM mutant virus by sequencing. (**E**) There was no difference in the one-step growth curve between the CHIKV TF ΔPBM and the CHIKV WT. Huh7.5.1 cells were infected with either the CHIKV TF ΔPBM (MOI = 3) or the CHIKV WT (MOI = 3). The titers of the supernatants from the indicated times were measured in Vero cells. (**F**) There was no difference in the multiple-step growth curve between the CHIKV TF ΔPBM and the CHIKV WT. Huh7.5.1 cells were infected with either the CHIKV TF ΔPBM (MOI = 0.01) or the CHIKV WT (MOI = 0.01). The supernatants were collected at the indicated times, and the plaque assay was performed in Vero cells. (**G**) KEGG enrichment analysis of the RNA sequencing of cells infected with CHIKV WT compared with the cells infected with CHIKV TF ΔPBM. HeLa cells were infected with CHIKV WT (MOI = 3) or CHIKV TF ΔPBM (MOI = 3) for 12 h, and the total RNA was isolated for RNA sequencing. (**H**) TF reduces the surface level of GLUT1 in HeLa cells. HeLa cells transfected with Flag-GLUT1 and either GFP-TF, GFP-TF ΔPBM mutant, or GFP were stained with an antibody against Flag (red). Scale bar: 10 μm. (**I**) The relative GLUT1 intensity was normalized by quantifying at least 13 cells using ImageJ. Statistical significance was determined using unpaired two-tailed multiple *t*-tests. ns, *P* > 0.05; ****, *P* < 0.0001. (**J**) CHIKV reduces the surface level of GLUT1, which could be recovered by Merafloxacin. HeLa cells infected with CHIKV or co-treated with 20 μM merafloxacin were stained with antibodies against Flag (red) and TF (green). Scale bar: 10 μm. (**K**) The relative GLUT1 intensity was normalized by quantifying at least 10 cells using ImageJ. Statistical significance was determined using unpaired two-tailed multiple *t*-tests. ns, *P* > 0.05; ****, *P* < 0.0001. (**L**) CHIKV TF impacts the surface level of GLUT1 in HeLa cells. HeLa cells infected with CHIKV TF ΔPBM mutant virus and transfected with Flag-GLUT1 for 24 h were stained with antibodies against Flag (red) and TF (green). Scale bar: 10 μm. (**M**) The relative GLUT1 intensity was normalized by quantifying at least 20 cells using ImageJ. Statistical significance was determined using unpaired two-tailed multiple *t*-tests. ns, *P* > 0.05; ****, *P* < 0.0001.

Chikungunya virus (CHIKV) is an arthritogenic alphavirus transmitted primarily by *Aedes* mosquitoes, responsible for significant morbidity across tropical and subtropical regions worldwide (1). CHIKV protein TF is derived from the 6K gene through a −1 ribosomal frame-shifting mechanism (2). An analysis of the CHIKV TF protein sequence identified STV as a class I PDZ-binding motif (PBM) at the C-terminus (Fig. 1A), which is recognized for its interaction with the PDZ domain of SNX27. SNX27 is pivotal for the endocytic recycling of cargos from endosomes to the plasma membrane (3). Then, we created a TF ΔPBM mutant to assess the interaction between TF and the SNX27 PDZ domain. Results from GST pulldown assays indicated that TF interacted with the SNX27 PDZ domain, whereas the ΔPBM mutant lost this interaction capability (Fig. 1B). The interaction with the retromer complex is characterized by the direct binding of the SNX27 PDZ domain to Vps26, enhancing the specificity for PDZ-binding motifs and promoting cargo recycling (4). Previously, we found that the spike protein from severe acute respiratory syndrome coronavirus 2 (SARS-CoV-2), which contained a PBM, inhibited SNX27-mediated endocytic recycling by disrupting the interaction between SNX27 and Vps26A (5). To investigate whether TF affected SNX27’s recycling activity through its PBM, we co-expressed TF, TF ΔPBM, and SNX27 subunit Vps26A in 293T cells. The GST pulldown assay demonstrated that TF inhibited the binding of Vps26A to SNX27 PDZ domain (Fig. 1C). However, the ΔPBM mutant did not exhibit this effect, suggesting that TF directly impeded SNX27’s interaction with Vps26A, thereby affecting SNX27’s binding to its cargo.

To gain insights into the role of the PBM in TF, we constructed a CHIKV TF ΔPBM mutant virus (Fig. 1D), which exhibited growth curves in both multiple-step and one-step assays similar to those of CHIKV WT (Fig. 1E and F). We then performed RNA sequencing on cells infected with either CHIKV WT or the CHIKV TF ΔPBM mutant virus. Interestingly, Kyoto Encyclopedia of Genes and Genomes (KEGG) analyses revealed that cells infected with CHIKV TF ΔPBM exhibited alterations in genes and metabolic pathways related to glycolysis and gluconeogenesis (Fig. 1G). Those results are consistent with the phenomena of SNX27 deficiency (3).

Glucose transporter type 1 (GLUT1) is a well-known cargo in SNX27-mediated endocytic recycling from endosomes to the plasma membrane (3). To evaluate TF’s impact on plasma membrane localization of SNX27 cargo GLUT1, we co-expressed GLUT1 with TF or TF ΔPBM in HeLa cells. Compared with the GFP control, the expression of TF decreased surface GLUT1 levels, whereas the TF ΔPBM mutant did not show this reduction (Fig. 1H and I). This indicates that TF impairs SNX27-mediated GLUT1 recycling, leading to diminished plasma membrane levels of GLUT1. Further validation involved overexpressing GLUT1 in HeLa cells, followed by CHIKV infection, and subsequent TF inhibition using −1 ribosomal frame-shifting inhibitor merafloxacin (6). Immunofluorescence analysis showed that CHIKV infection reduced GLUT1 surface levels, which were restored upon merafloxacin treatment (Fig. 1J and K). Interestingly, the levels of GLUT1 at the plasma membrane remained unchanged in cells infected with the TF ΔPBM mutant compared with mock-infected cells (Fig. 1L and M). These findings suggest that TF can inhibit the binding of SNX27 to GLUT1 via its PBM, thereby impacting the SNX27-mediated recycling of GLUT1.

**Fig 2:**
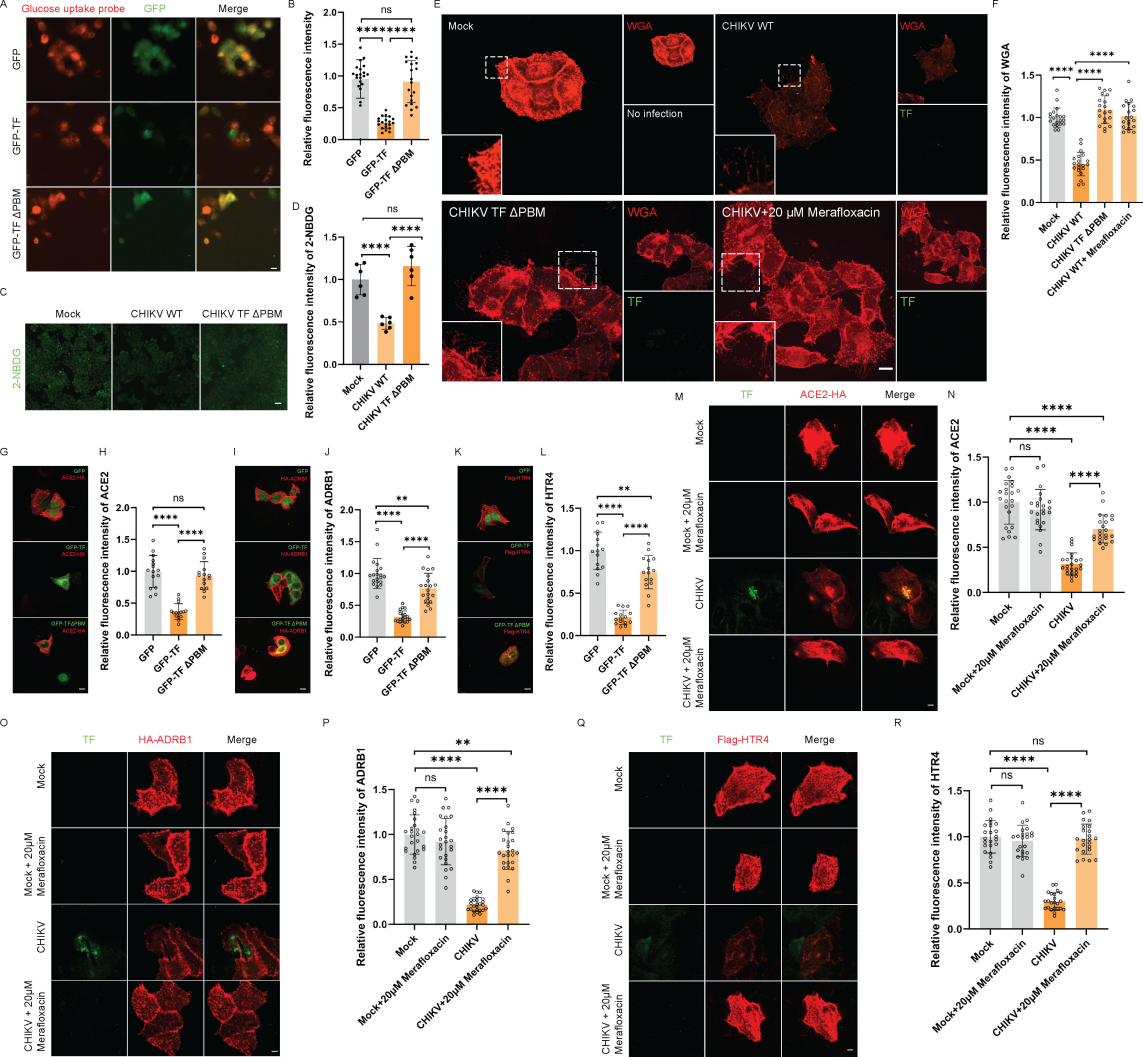
PBM is required for CHIKV-mediated inhibition of glucose uptake and surface levels of other SNX27 cargo proteins. (**A**) TF inhibits glucose uptake via PBM. HeLa cells were transfected with GFP, GFP-TF, or GFP-TF ΔPBM, and glucose uptake levels were measured 24 h later using a glucose uptake probe-red (UP03). Scale bar: 50 μm. (**B**). Statistical analysis of the fluorescence intensity of the glucose uptake probe by quantifying at least 20 cells using ImageJ from panel **A**. Statistical significance was determined using unpaired two-tailed multiple *t*-tests. ns, *P* > 0.05; ****, *P* < 0.0001. (**C**) PBM in TF is required for the reduction of glucose uptake mediated by CHIKV. HeLa cells infected with CHIKV WT or the mutant virus (MOI = 3) were incubated at 37°C with 500 μM 2-NBDG for 3 h following a 12-h infection period. The 2-NBDG fluorescence was measured using an M7000 confocal microscope. Scale bar, 50 μm. (**D**) Relative 2-NBDG intensity was normalized by quantifying at least six fields of view using ImageJ from panel **C**. Statistical significance was determined using unpaired two-tailed multiple *t*-tests. ns, *P* > 0.05; ****, *P* < 0.0001. (**E**) GlcNAc level increased when TF was inhibited in cells infected with CHIKV. HeLa cells were infected with CHIKV TF ΔPBM mutant virus or mock infection for 10 h. CHIKV-infected HeLa cells were treated with 20 μM merafloxacin or mock treatment for 10 h. WGA (red) indicates GlcNAc level, and TF is shown in green. Scale bar, 10 μm. (**F**) Relative WGA intensity was normalized by quantifying at least 20 cells using ImageJ from panel **E**. ****, *P* < 0.0001 (unpaired two-tailed multiple *t*-tests). (**G**) TF reduces the surface level of ACE2. HeLa cells transfected with ACE2-HA and either GFP-TF, GFP-TF ΔPBM mutant, or GFP were stained with an anti-HA antibody (in red). Scale bar: 10 μm. (**H**) The relative ACE2 intensity from panel **G** was normalized by quantifying at least 20 HeLa cells using ImageJ. Statistical significance was determined using unpaired two-tailed multiple *t*-tests. Statistical significance was determined using unpaired two-tailed multiple *t*-tests. ns, *P* > 0.05; ****, *P* < 0.0001. (**I**) TF reduces the surface level of ADRB1. HeLa cells transfected with HA-ADRB1 and either GFP-TF, GFP-TF ΔPBM mutant, or GFP were stained with an anti-HA antibody (in red). Scale bar: 10 μm. (**J**) The relative ADRB1 intensity from panel **I** was normalized by quantifying at least 20 HeLa cells using ImageJ. Statistical significance was determined using unpaired two-tailed multiple *t*-tests. Statistical significance was determined using unpaired two-tailed multiple *t*-tests. ∗∗, *P* < 0.01; **** *P* < 0.0001. (**K**) TF reduces the surface level of HTR4. HeLa cells transfected with Flag-HTR4 and either GFP-TF, GFP-TF ΔPBM mutant, or GFP were stained with an anti-Flag antibody (in red). Scale bar: 10 μm. (**L**) The relative HTR4 intensity from panel **K** was normalized by quantifying at least 20 HeLa cells using ImageJ. Statistical significance was determined using unpaired two-tailed multiple *t*-tests. **, *P* < 0.01; ****, *P* < 0.0001. (**M**) CHIKV reduces the surface level of ACE2, which could be recovered by merafloxacin. HeLa cells infected with CHIKV or co-treated with 20 μM merafloxacin were stained with anti-HA (in red) and anti-TF (in green) antibodies. Scale bar: 10 μm. (**N**) The relative ACE2 intensity from panel **M** was normalized by quantifying at least 20 HeLa cells using ImageJ. Statistical significance was determined using unpaired two-tailed multiple *t*-tests. ns, *P* > 0.05; ****, *P* < 0.0001. (**O**) CHIKV reduces the surface level of ADRB1, which could be recovered by merafloxacin. HeLa cells infected with CHIKV or co-treated with 20 μM merafloxacin were stained with anti-HA (in red) and anti-TF (in green) antibodies. Scale bar: 10 μm. (**P**) The relative ADRB1 intensity from panel **O** was normalized by quantifying at least 20 HeLa cells using ImageJ. Statistical significance was determined using unpaired two-tailed multiple *t*-tests. ns, *P* > 0.05; **, *P* < 0.01; ****, *P* < 0.0001. (**Q**) CHIKV reduces the surface level of HTR4, which could be recovered by merafloxacin. HeLa cells infected with CHIKV or co-treated with 20 μM merafloxacin were stained with anti-Flag (in red) and anti-TF (in green) antibodies. Scale bar: 10 μm. (**R**) The relative Flag intensity from panel **Q** was normalized by quantifying at least 20 HeLa cells using ImageJ. Statistical significance was determined using unpaired two-tailed multiple *t*-tests. ns, *P* > 0.05; ****, *P* < 0.0001.

Given that GLUT1 is the primary glucose transporter (7), we investigated whether TF could lower glucose uptake via its PBM. In HeLa cells overexpressing TF and the TF Δ PBM mutant, we assessed the impact of TF on glucose uptake using a glucose uptake detection probe. The results demonstrated a decrease in glucose uptake levels in cells overexpressing TF, while the TF Δ PBM mutant exhibited no effect on glucose uptake (Fig. 2A and B). Moreover, in HeLa cells infected with CHIKV WT, and CHIKV TF Δ PBM mutants, glucose uptake levels were measured using the 2-NBDG probe. The result indicated a notable reduction in glucose uptake in cells infected with CHIKV WT (Fig. 2C and D). In contrast, glucose uptake levels remained unaffected in cells infected with the CHIKV TF Δ PBM virus compared with mock infection (Fig. 2C and D). These findings suggest that TF can reduce glucose uptake through its PBM.

Elevated glucose enhances uridine diphosphate N-acetylglucosamine (UDP-GlcNAc) production, increasing GlcNAc levels through a dynamic modification process (8). Wheat germ agglutinin (WGA) staining reflects GlcNAc levels (9). We examined the effect of TF on GlcNAc by infecting HeLa cells with CHIKV or using merafloxacin during CHIKV infection. CHIKV infection reduced GlcNAc level (Fig. 2E and F). In contrast, CHIKV TF ΔPBM or merafloxacin treatment during CHIKV infection exhibited enhanced GlcNAc levels compared with the CHIKV WT group (Fig. 2E and F), suggesting that intact TF inhibits GlcNAc level. These resultss imply that TF reduces GlcNAc levels by inhibiting the endocytic recycling of GLUT1.

While our results indicate that the PBM is necessary for the interaction between TF and the SNX27 PDZ domain (Fig. 1B), we also recognize that other regions of the TF protein may play a contributory role in this interaction. Therefore, although the PBM is both necessary and sufficient for the observed functional inhibition, we cannot exclude the potential involvement of other protein domains. Further studies could investigate these additional contributions to provide a more comprehensive understanding of the mechanisms underlying TF’s interaction with SNX27.

Serotonin (5-hydroxytryptamine [5-HT]) receptor 4 (HTR4), the beta-1 adrenergic receptor (ADRB1), and angiotensin-converting enzyme 2 (ACE2) are known to be cargoes for SNX27-mediated endocytic recycling (10, 11). To assess the impact of TF on the plasma membrane localization of these proteins, we co-expressed ACE2, ADRB1, or HTR4 alongside either TF or the TF ΔPBM mutant in HeLa cells. Compared with the GFP control, the expression of TF significantly decreased the surface levels of ACE2, ADRB1, and HTR4, while the TF ΔPBM mutant did not display this reduction (Fig. 2G through L).

Additionally, we overexpressed ACE2, ADRB1, or HTR4 in HeLa cells, followed by CHIKV infection and subsequent inhibition of TF production using merafloxacin. CHIKV infection led to a substantial decrease in the surface levels of ACE2, ADRB1, and HTR4, which were restored upon treatment with merafloxacin (Fig. 2M through R). These findings suggest that TF inhibits the SNX27-mediated recycling of ACE2, ADRB1, and HTR4.

Typically, CHIKV-related disease presents with a triad of symptoms: fever, arthralgia, and rash. However, there has been an increase in reports of other clinical manifestations, such as cognitive dysfunction (12), depression (12), heart failure (13), and hypertension (13). GLUT1 serves as the primary energy transporter in the brain. Reduced GLUT1 protein levels cause dystonia and GLUT1 deficiency syndrome 1 (GLUT1DS1), which is characterized by ataxia, sleep disturbances, and muscle spasticity (7). ACE2 inhibition is associated with hypertension and other diseases (14), whereas ADRB1 inhibition can lead to heart failure and hypertension (15). Additionally, inhibition of HTR4 is known to contribute to depression and cognitive impairment (16). Our current findings support a model in which these symptoms arise, in part, from the deficiency of GLUT1, ACE2, ADRB1, and HTR4 due to the action of CHIKV protein TF. To function properly, GLUT1, ACE2, ADRB1, and HTR4 are transported from endosomes to the plasma membrane by SNX27 and the retromer complex. However, during CHIKV infection, TF suppresses the endocytic recycling of GLUT1, ACE2, ADRB1, and HTR4 by inhibiting the interaction between SNX27 and Vps26A through its PBM. As a result, the surface levels of GLUT1, ACE2, ADRB1, and HTR4 are diminished, potentially contributing to cognitive dysfunction, depression, heart failure, and hypertension.

In summary, our findings reveal that CHIKV protein TF inhibits SNX27-mediated endocytic recycling of GLUT1, crucial for glucose homeostasis at the plasma membrane. By disrupting SNX27’s interaction with the retromer complex through its PBM, TF significantly reduces GLUT1 recycling and glucose uptake, illustrating how CHIKV manipulates host cell metabolism to enhance its pathogenicity. This dysregulation extends to other receptors, including ACE2, ADRB1, and HTR4, potentially leading to a variety of clinical manifestations, such as neurological deficits, cardiovascular issues, and metabolic disorders. These results underscore the complex interplay between CHIKV and host cellular pathways, highlighting the need for further research into the molecular mechanisms at play and the development of targeted therapeutic interventions to restore normal receptor trafficking and metabolic balance, thereby improving outcomes in CHIKV-related diseases.

## Data Availability

The authors confirm that all data underlying the findings are fully available without restriction. RNA sequencing data are uploaded in Gene Expression Omnibus (GEO, https://www.ncbi.nlm.nih.gov/geo/) with the accession number GSE299288.
